# Cracking the code of a coastal oil yeast: the genome of *Rhodotorula pacifica* INDKK

**DOI:** 10.1128/mra.00317-25

**Published:** 2025-08-27

**Authors:** Kiran Kumar Kukkala, Preeti Yadav, Ashish Kumar, Pannaga Pavan Jutur, Debarati Paul, Naseem A. Gaur

**Affiliations:** 1 Yeast Biofuel Group, International Centre for Genetic Engineering and Biotechnology 18470, New Delhi, India; 2 Omics of Algae Group, International Centre for Genetic Engineering and Biotechnology 18470, New Delhi, India; 3 Amity Institute of Biotechnology, Amity University Uttar Pradesh 663345, Noida, Uttar Pradesh, India; Rochester Institute of Technology, Rochester, New York, USA

**Keywords:** genome assembly, Illumina, nanopore, oleaginous yeast, contigs, lipids, *Rhodotorula*, annotation, telomere, *pacifica*

## Abstract

*Rhodotorula pacifica* INDKK, an oleaginous red yeast isolated from mangrove forest soil, efficiently metabolizes biomass hydrolysates and accumulates lipids of more than 60% of its dry weight. Hybrid *de novo* genome assembly (Illumina and Oxford Nanopore) produced 24 scaffolds comprising a 21.05 Mb nuclear and ~26 kb mitochondrial genome.

## ANNOUNCEMENT

*Rhodotorula pacifica* INDKK is an oleaginous red yeast isolated from Coringa mangrove forest, Kakinada, Andhra Pradesh (16.83139°N–82.33667°E), India. This strain shows strong potential for low-cost lipid and carotenoid production due to its ability to utilize both pentose and hexose sugars from lignocellulosic hydrolysates and higher tolerance to pretreatment-derived inhibitors (1, 2). Genome sequencing of *R. pacifica* INDKK was initiated for elucidating the metabolic and regulatory pathways related to lipid, carotenoid, and other valuable products with the aim of achieving increased production rates by employing genome engineering.

The strain was isolated by the serial dilution method and cultured in YPD medium (10 g/L yeast extract, 20 g/L peptone, amd 20 g/L dextrose) at 30°C and 200 rpm. Genomic DNA was extracted using enzymatic lysis and the CTAB (Cetyl Trimethyl Ammonium Bromide) method (3). The quality of the extracted genomic DNA was assessed by NanoDrop and agarose gel electrophoresis. The whole-genome sequencing was performed by combining short-read Illumina and long-read Oxford Nanopore platforms. Illumina libraries were prepared by using 100 ng of genomic DNA (QIASeq FX DNA Library Kit) and sequenced on a HiSeq X Ten (2 × 150 bp) platform. Nanopore libraries (600 ng DNA) were prepared using EXP-NBD114 and sequenced on a GridION X5 platform with R9.4 flow cell.

Oxford Nanopore reads were filtered with NanoFilt v2.6.0, and Illumina reads were trimmed using Fastp and assessed with FastQC v0.11.9. A hybrid *de novo* assembly was generated using MaSuRCA v4.0.3, followed by removal of haplotigs and duplications via Purge_dups v1.2.6. A mitochondrial contig identified via BLASTn was excluded to prepare an exclusively nuclear genome assembly. RepeatModeler v2.0.6 and RepeatMasker v4.1.9 were used for repeat modeling and soft-masking. Tandem Repeats Finder identified canonical telomeric repeats (TTAGGG) at both ends of scaffolds 5, 9, and 10, indicating near chromosome-level assembly. Scaffolds 2, 20, and 22 had canonical repetitions on only one end, while Scaffold-1 contained several internal tandem telomeric-like repeats, indicating subtelomeric or interstitial telomeric repeats. BUSCO v5.8.0 analysis indicated 94.4% complete orthologs, comprising 88.9% single-copy, 5.5% duplicated, 1% fragmented, and 4.6% missing genes. QUAST v5.3.0 was used to assess additional assembly metrics (Table 1). The draft genome sequence of *R. pacifica* comprises 24 scaffolds with a total genome size of 21.05 Mb and 59.4% GC content, which is consistent with earlier studies involving oleaginous red yeasts (4, 5).

**TABLE 1:** Table showing the genome assembly statistics of *R. pacifica* INDKK

Parameter	Number
Total no. of scaffolds	24
N’s per 100 kbp	16
No. of scaffolds (≥1,000 bp)	24
Total length	21,056,800
Largest contig	2,764,732
GC %	59.40
N50 value	1,305,572
N90 value	669,876
Assembly level	Scaffold
Genome coverage	360×
L50	6
L90	15
No. of N’s per 100 kbp	16.19

Paired-end RNA-seq reads were aligned to the soft-masked genome using HISAT2 v2.2.1. Splice junctions were identified using GeneMark-ET v4.65 in fungal mode and used by AUGUSTUS v3.4.0 under BRAKER2 for ab initio gene prediction, which predicted 7,809 coding sequences. Functional annotation using EggNOG-mapper v5.0 assigned GO terms to 5,735 predicted proteins.

## Data Availability

The *R. pacifica* INDKK whole-genome sequencing project has been deposited at DDBJ/EMBL/GenBank under the accession number CBDEHS030000000.3. The revised third version was analyzed and described in this paper.
